# Immunogenicity of a Trivalent Human Papillomavirus L1 DNA-Encapsidated, Non-Replicable Baculovirus Nanovaccine

**DOI:** 10.1371/journal.pone.0095961

**Published:** 2014-04-23

**Authors:** Hansam Cho, Hee-Jung Lee, Yoon-Ki Heo, Yeondong Cho, Yong-Dae Gwon, Mi-Gyeong Kim, Ki Hoon Park, Yu-Kyoung Oh, Young Bong Kim

**Affiliations:** 1 Department of Bio-industrial Technologies, Konkuk University, Seoul, Republic of Korea; 2 College of Pharmacy, Seoul National University, Seoul, Republic of Korea; Shanghai Medical College, Fudan University, China

## Abstract

Previously, we developed a non-replicating recombinant baculovirus coated with human endogenous retrovirus envelope protein (AcHERV) for enhanced cellular delivery of human papillomavirus (HPV) 16L1 DNA. Here, we report the immunogenicity of an AcHERV-based multivalent HPV nanovaccine in which the L1 segments of HPV 16, 18, and 58 genes were inserted into a single baculovirus genome of AcHERV. To test whether gene expression levels were affected by the order of HPV L1 gene insertion, we compared the efficacy of bivalent AcHERV vaccines with the HPV 16L1 gene inserted ahead of the 18L1 gene (AcHERV-HP16/18L1) with that of AcHERV with the HPV 18L1 gene inserted ahead of the 16L1 gene (AcHERV-HP18/16L1). Regardless of the order, the bivalent AcHERV DNA vaccines retained the immunogenicity of monovalent AcHERV-HP16L1 and AcHERV-HP18L1 DNA vaccines. Moreover, the immunogenicity of bivalent AcHERV-HP16/18L1 was not significantly different from that of AcHERV-HP18/16L1. In challenge tests, both bivalent vaccines provided complete protection against HPV 16 and 18 pseudotype viruses. Extending these results, we found that a trivalent AcHERV nanovaccine encoding HPV 16L1, 18L1, and 58L1 genes (AcHERV-HP16/18/58L1) provided high levels of humoral and cellular immunogenicity against all three subtypes. Moreover, mice immunized with the trivalent AcHERV-based nanovaccine were protected from challenge with HPV 16, 18, and 58 pseudotype viruses. These results suggest that trivalent AcHERV-HPV16/18/58L1 could serve as a potential prophylactic baculoviral nanovaccine against concurrent infection with HPV 16, 18, and 58.

## Introduction

Human papillomaviruses (HPV) are a heterogeneous group of double-stranded DNA viruses that cause malignant tumors of the anogenital tract, leading to cervical cancer, a common cancer accounting for approximately 12% of all cancers in women [1]. Over 100 different types of HPV, divided into low-risk and high-risk, have been identified [2]. The high-risk HPV types include HPV 16, 18, 31, 45, and 58. Among high-risk HPV types, HPV 16 and 18 predominate, accounting for more than 70% of cervical cancers. Currently available prophylactic HPV vaccines target high-risk types such as HPV 16 and 18 [3], [4]. HPV 58 is also clinically significant, especially in Asia, where it is the third-most prevalent HPV type among cervical cancers reported in Korea, Japan, and southern and eastern parts of China [5]. The larger share of disease burden of HPV 58 in Asia may reflect differences in host genetics as well as the oncogenicity of circulating variants. This unique pattern of epidemic HPV58 prevalence should be considered in the development of next-generation HPV vaccines [6].

Several experimental vaccines have been studied for their potential to generate neutralizing antibodies against HPV. Current vaccination approaches include virus-like particles, recombinant fusion proteins, recombinant fusion peptides, live recombinant bacteria and recombinant viruses [7]. DNA vaccines have received particular research attention as next-generation vaccines that may replace current subunit or live-attenuated vaccines. DNA vaccines offer several advantages compared to conventional vaccines, including relative stability and safety, capacity to induce cell-mediated immune responses and ease of manipulation. Moreover, they can be created using less complex production processes and are thus less expensive to produce on a large scale. Despite these advantages and initial high hopes, research progress in this area since the first report about two decades ago has been slow, with only a few DNA vaccines reaching clinical trials to date [8], [9]. One major limitation that has hampered the successful development of DNA vaccines is the intracellular delivery issue: because of their highly negative charge and large size, naked plasmid DNA cannot effectively penetrate the cell membrane [10], [11].

To improve the efficacy of DNA vaccine cellular delivery, researchers have investigated various nonviral and viral vectors. Nonviral cationic liposomes [12] and polymers [13] have been studied as delivery systems for plasmid DNA vaccines, and physical methods have been applied for introducing DNA into cells [14], [15]. Recombinant adenovirus [16] and vaccinia virus [17] have been investigated as delivery systems for antigen-encoding DNA. Although viral vectors have advantages over nonviral vector systems in terms of intracellular delivery efficacy, they suffer from at least two major drawbacks from the standpoint of clinical development. First, most viral vectors can be converted to pathogenic forms after replication. Second, viral vectors are immunogenic, limiting repeated dosing with DNA vaccines.

Overcoming the limitations of currently studied viral vectors requires the development of new viral vectors that do not replicate in human cells, which would eliminate the potential conversion to pathogenic forms and immunogenicity, thereby allowing repeated dosing with DNA vaccines [18]. We previously developed a viral DNA vaccine against HPV 16 using recombinant baculovirus [19]. The baculovirus system is advantageous because it does not support replication in mammalian cells while increasing the efficiency of gene delivery [20]–[23]. To enhance delivery of the HPV L1 gene into human hosts, we previously constructed a recombinant baculovirus containing the envelope glycoprotein of human endogenous retrovirus (AcHERV). These AcHERV-based vaccines induced strong humoral and cellular immune responses in mice as well as pig models [24], [25].

In this study, we constructed a trivalent AcHERV-based nanovaccine encoding HPV 16L1, 18L1, and 58L1 genes (AcHERV-HP16/18/58L1). Here, we report the immunogenicity of the trivalent nanovaccine and demonstrate its capacity to provide protection against pseudotype HPV 16, 18, and 58.

## Results

### Construction of AcHERV-based multivalent nanovaccines

**Figure 1:**
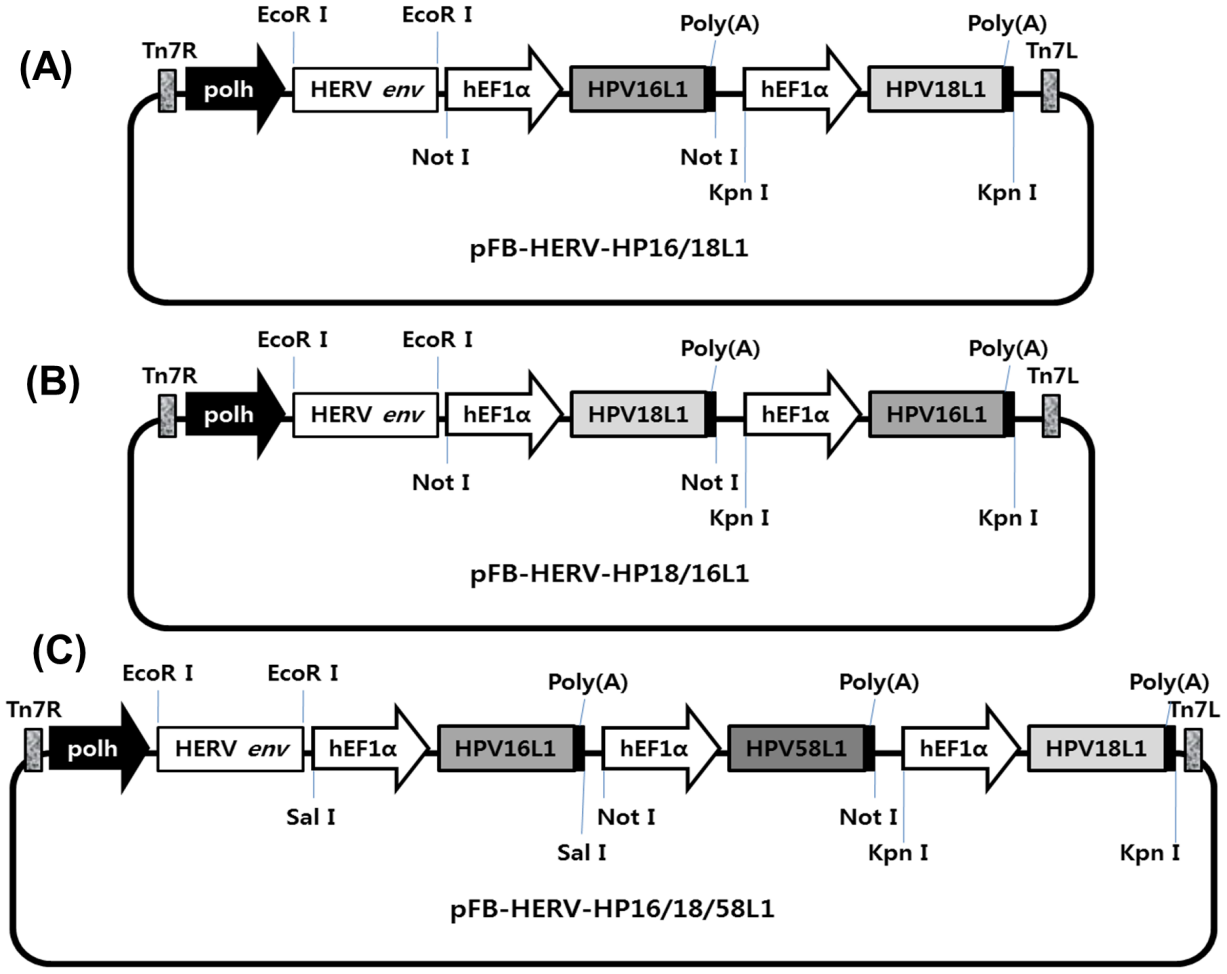
Scheme of transfer plasmids for construction of recombinant baculoviruses. The structure of the transfer plasmid was derived from pFastBac1. In all plasmids, Polh (polyhedron promoter of baculovirus) was used as a promoter of HERV *env* gene. As a polycistronic promoter, hEF1α was used for HPV16, 18, and 58L1 genes. For each plasmid, poly(A) (polyadenylation signal) was inserted at the end of each target gene. (A) pFB-HERV-HP16/18L1 was constructed by putting HPV 16L1 gene ahead of HPV 18L1. (B) pFB-HERV-HP18/16L1 was constructed by putting HPV 18L1 gene ahead of HPV 16L1. (C) pFB-HERV-HP16/18/58L1 was constructed by inserting HPV 58L1 gene between HPV 16L1 to HPV 18 L1 gene.

**Figure 2:**
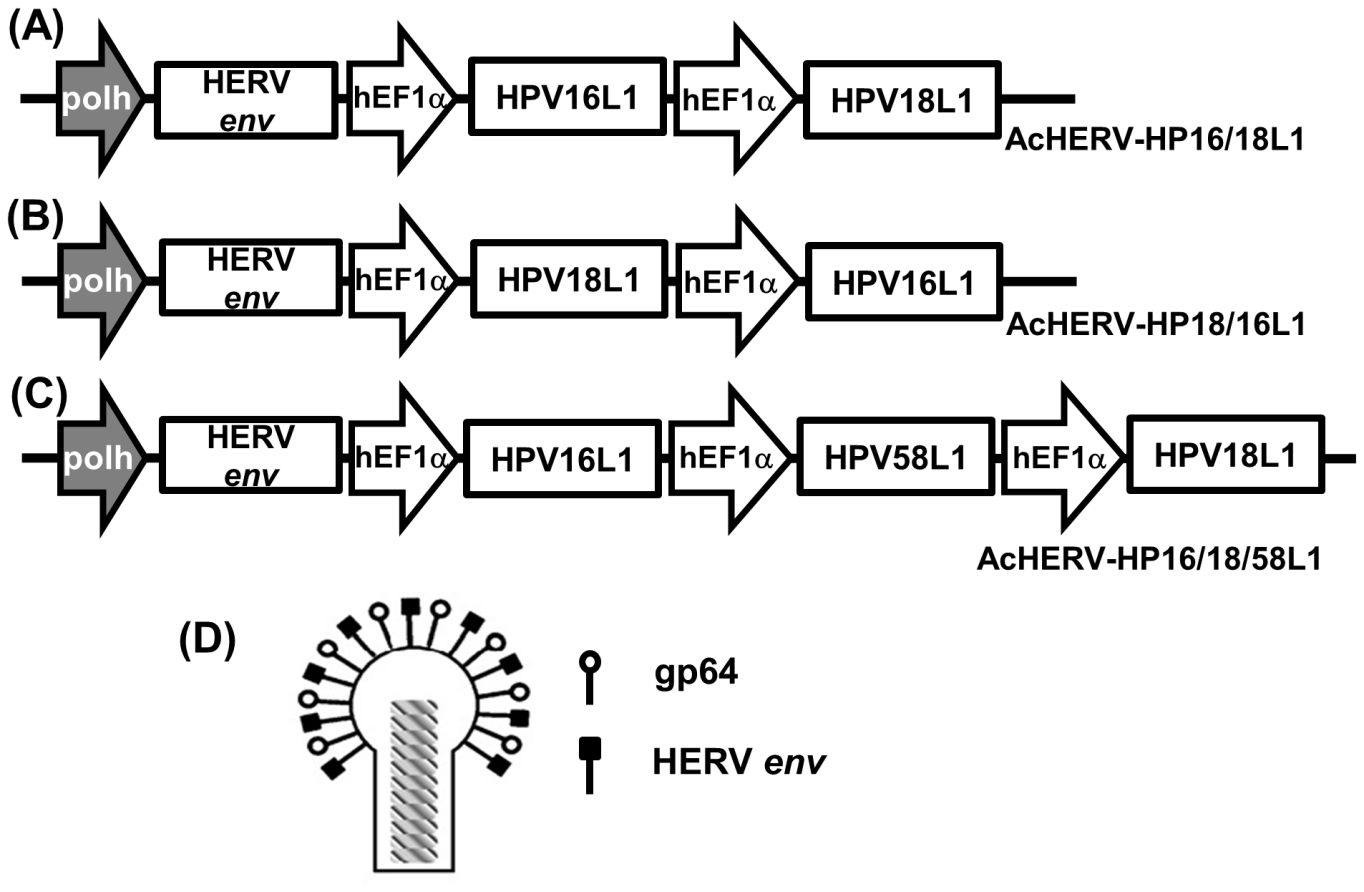
Construction of recombinant baculoviruses encoding HPV 16L1 or HPV 18L1 or HPV 58L1. Bivalent AcHERV-HP16/18L1 (A), AcHERV-HP18/16L1 (B), and trivalent AcHERV-HP16/18/58L1 (C) were constructed to contain the hEF1α promoter controlling L1 protein expression and the Polh promoter controlling HERV envelope expression. (D) AcHERV-based recombinant baculoviruses were generated using the Bac-to-Bac expression system.

**Figure 3:**
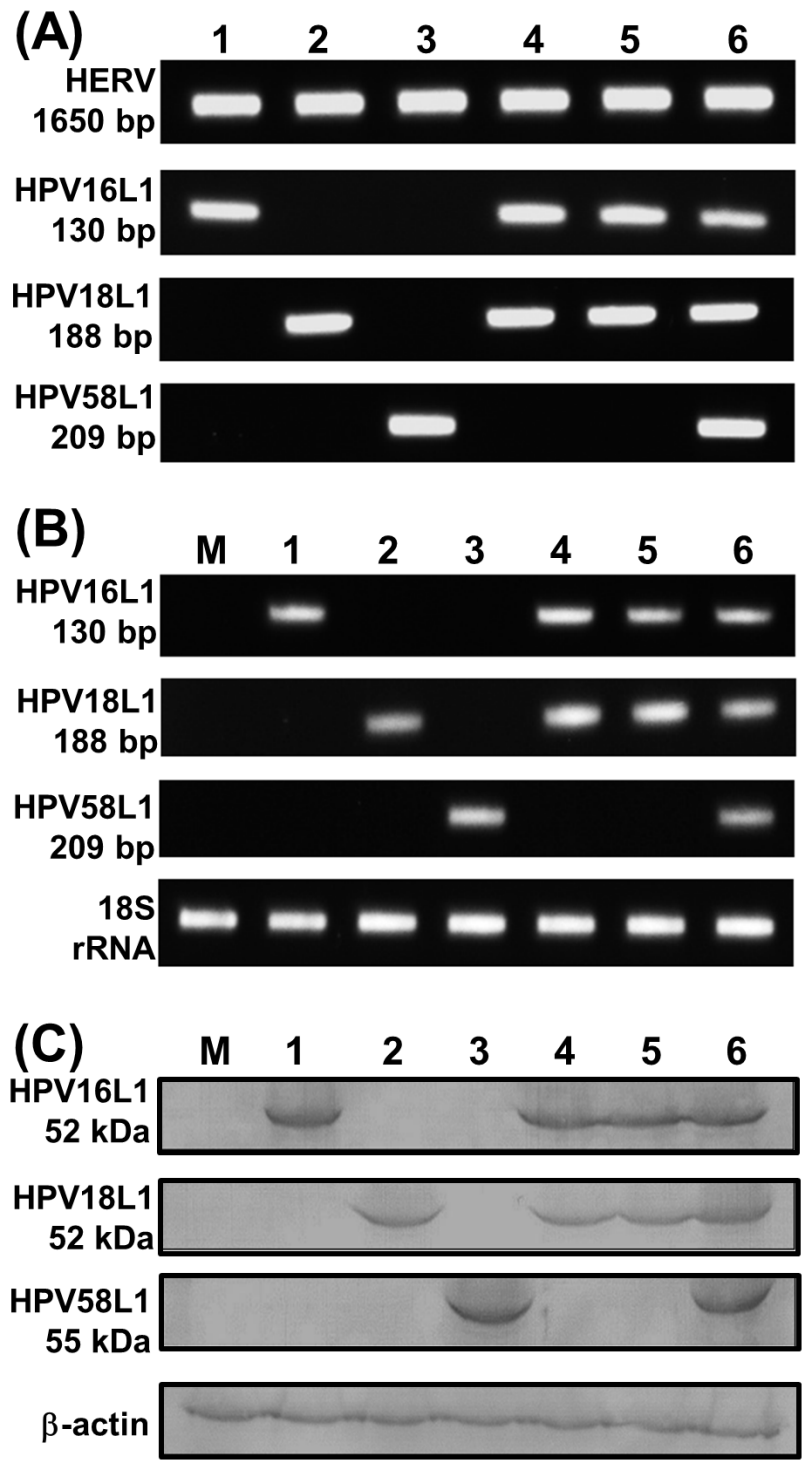
Chracterization of AcHERV-based nanovaccines and expression of HPV L1s. AcHERV-based nanovaccines were generated by amplification of HERV *env* and HPVL1 genes from viral DNA (A) for recombinant AcHERV-HP16L1 (lane 1), AcHERV-HP18L1 (lane 2), AcHERV-HP58L1 (lane 3), AcHERV-HP16/18L1 (lane 4), AcHERV-HP18/16L1 (lane 5), and AcHERV-HP16/18/58 L1 (lane 6) using specific PCR primers. Expression of HPVL1 genes was performed RT-PCR (B) and western blot (C) in 293TT cells infected with for recombinant AcHERV-HP16L1 (lane 1), AcHERV-HP18L1 (lane 2), AcHERV-HP58L1 (lane 3), AcHERV-HP16/18L1 (lane 4), AcHERV-HP18/16L1 (lane 5), and AcHERV-HP16/18/58 L1 (lane 6). Lane M is uninfected 293TT cells.

To test the feasibility of the AcHERV vector as a multivalent DNA nanovaccine system, we first constructed transfer plasmids (Fig. 1) and then recombinant baculoviruses (Fig. 2). A schematic of transfer plasmids is shown in Figure 1. All transfer plasmids were constructed to express the HERV *env* gene under the control of the polyhedron promoter, and HPV L1 under the control of the hEF1-α promoter system. The transfer plasmids, pFB-HERV-HP16/18L1 (Fig. 1A), pFB-HERV-HP18/16L1 (Fig. 1B), and pFB-HERV-HP16/18/58L1 (Fig. 1C) were used to construct bivalent AcHERV-HP16/18L1 (Fig. 2A), AcHERV-HP18/16L1 (Fig. 2B), and trivalent AcHERV-HP16/18/58L1 (Fig. 2C), respectively. All recombinant baculoviruses were designed to express HERV *env* (Fig. 2D). The construction of recombinant baculoviruses was confirmed by PCR using primers specific for HPV16L1, HPV18L1, HPV58L1, and HERV *env*. PCR products showed the presence of HERV *env* and the unique presence of the HPV16L1, HPV18L1, or HPV58L1 gene in the corresponding AcHERV-based multivalent nanovaccine (Fig. 3A).

RT-PCR analyses performed following infection of 293TT mammalian cells with AcHERV-based multivalent nanovaccines showed expression of HPV 16L1, 18L1, or 58L1 mRNA (Fig. 3B). Western blotting showed that cells transduced with bivalent or trivalent AcHERV-based nanovaccines expressed HPV 16L1 and 18L1 proteins with an approximate molecular weight of ∼52 kDa (Fig. 3C). Both monovalent AcHERV-HP58L1-treated cells and trivalent AcHERV-HP16/18/58L1-treated cells showed expression of HPV 58L1 protein with a molecular weight of ∼55 kDa (Fig. 3C).

### Humoral immune responses following administration of AcHERV-based multivalent nanovaccines

**Figure 4:**
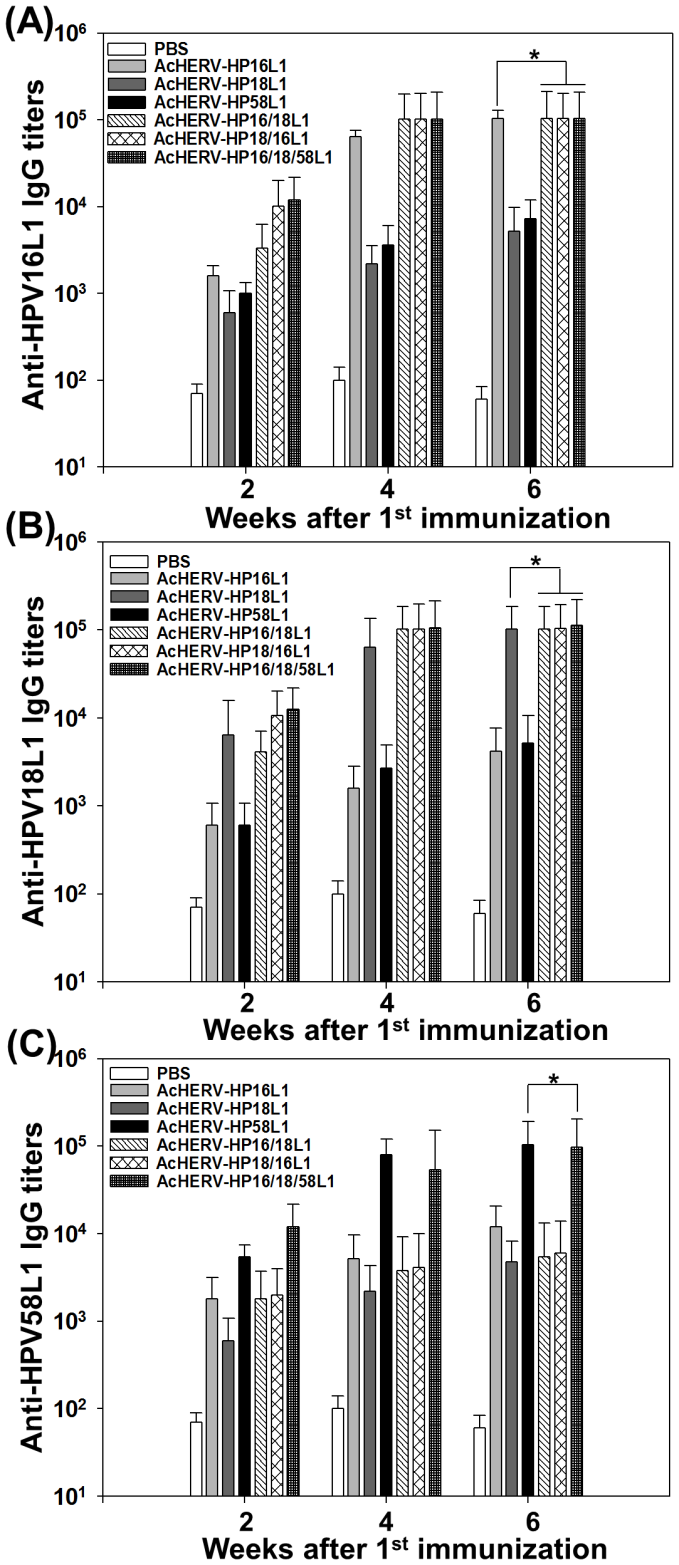
Induction of HPV type-specific L1s IgG antibodies in mouse serum. BALB/c mice were intramuscularly injected with PBS or 1×10^7^ PFU of AcHERV-based nanovaccines. Antigen specific IgG antibodiy titers against HPV 16L1 (A), HPV 18L1 (B), or HPV 58L1 (C) in murine sera were determined by ELISA. * p<0.05 (ANOVA and Student-Newman-Keuls test): Bivalent and trivalent groups were compared with each monovalent group.

The humoral immune responses induced by bivalent and trivalent AcHERV-based nanovaccines were similar to those induced by the monovalent AcHERV-based AcHERV-HP16L1, AcHERV-HP18L1, and AcHERV-HP58L1 nanovaccines. The immunization schedules for seven groups are summarized in Table 1. Six weeks after the first administration, the levels of serum IgG antibodies specific for HPV 16 (Fig. 4A) were not significantly different among groups treated with AcHERV-HP16L1, AcHERV-HP16/18L1, AcHERV-HP18/16L1, or AcHERV-HP16/18/58L1. Similarly, the levels of serum IgG antibodies specific for HPV 18 were not significantly different among groups treated with AcHERV-HP18L1, AcHERV-HP16/18L1, AcHERV-HP18/16L1, or AcHERV-HP16/18/58L1 (Fig. 4B). Figure 4C shows that the levels of serum IgG antibodies specific for HPV58 were not significantly different between groups receiving AcHERV-HP58L1 or AcHERV-HP16/18/58L1.

**Table 1:** Immunization schedules.

	Immunization (2-weeks intervals)	
Groups	Vaccine construct	No. of mice
Group 1	PBS	15
Group 2	AcHERV-HP16L1	8
Group 3	AcHERV-HP18L1	8
Group 4	AcHERV-HP58L1	8
Group 5	AcHERV-HP16/18L1	8
Group 6	AcHERV-HP18/16L1	8
Group 7	AcHERV-HP16/18/58L1	8

Each group (8 mice per group) was immunized three times at 2-week intervals with AcHERV-HPL1s (AcHERV-HP16L1 or AcHERV-HP18L1 or AcHERV-HP58L1 or AcHERV-HP16/18L1 or AcHERV-HP18/16L1 or AcHERV-HP16/18/58L1) at a dose of 10^7^ PFU per mouse by intramuscular route. Two weeks after the immunization, mice sera and vaginal washings were collected.

**Figure 5:**
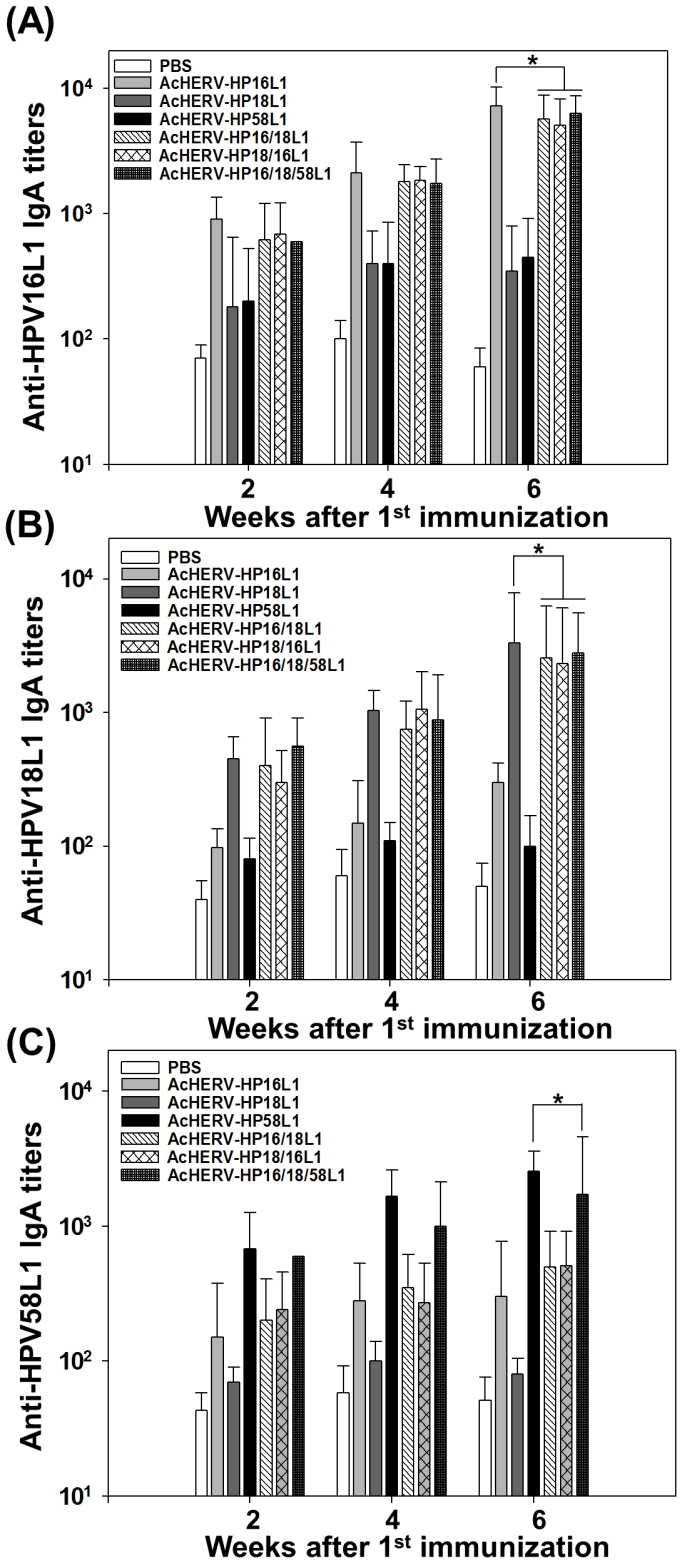
Induction of HPV type-specific L1s IgA antibodies in vaginal washes. BALB/c mice were intramuscularly injected with PBS or 1×10^7^ PFU of AcHERV-based nanovaccines. Antigen specific IgA antibodiy titers against HPV 16L1 (A) or HPV 18L1 (B) or HPV 58L1 (C) in murine vaginal washes were determined by ELISA. * p<0.05 (ANOVA and Student-Newman-Keuls test): Bivalent and trivalent groups were compared with each monovalent group.

Because mucosal antibodies are crucial in protecting against sexually transmitted infections and form the first line of defense against such infectious agents [26], the secretory IgA response is an import marker of mucosal immunity. Following administration of AcHERV-based nanovaccines, vaginal IgAs specific for HPV 16 (Fig. 5A), HPV 18 (Fig. 5B), and HPV 58 (Fig. 5C) were induced with kinetics similar to those of serum IgG, reaching a high level 6 weeks after the first administration. Moreover, there was no significant difference in the levels of vaginal anti-HPV16L1-specific (Fig. 5A) or anti-HPV18L1-specific (Fig. 5B) IgA antibodies induced by bivalent or trivalent AcHERV-based nanovaccines and monovalent AcHERV-HP16L1 or AcHERV-HP18L1.

To test whether gene expression levels were affected by the order of gene insertion, we compared the efficacy of the bivalent AcHERV-based nanovaccines, AcHERV-HPV16/18L1 and AcHERV-HPV18/16L1. Regardless of the position of the two genes, bivalent AcHERV DNA vaccines induced levels of HPV16L1-specific IgG (Fig. 4A) and IgA (Fig. 5A) antibodies similar to those induced by AcHERV-HP16L1. Moreover, bivalent AcHERV DNA vaccines induced levels of HPV18L1-specific IgG (Fig. 4B) and IgA (Fig. 5B) antibodies similar to those induced by AcHERV-HP18L1.

### Induction of neutralizing antibodies after administration of AcHERV-based multivalent nanovaccines

**Figure 6:**
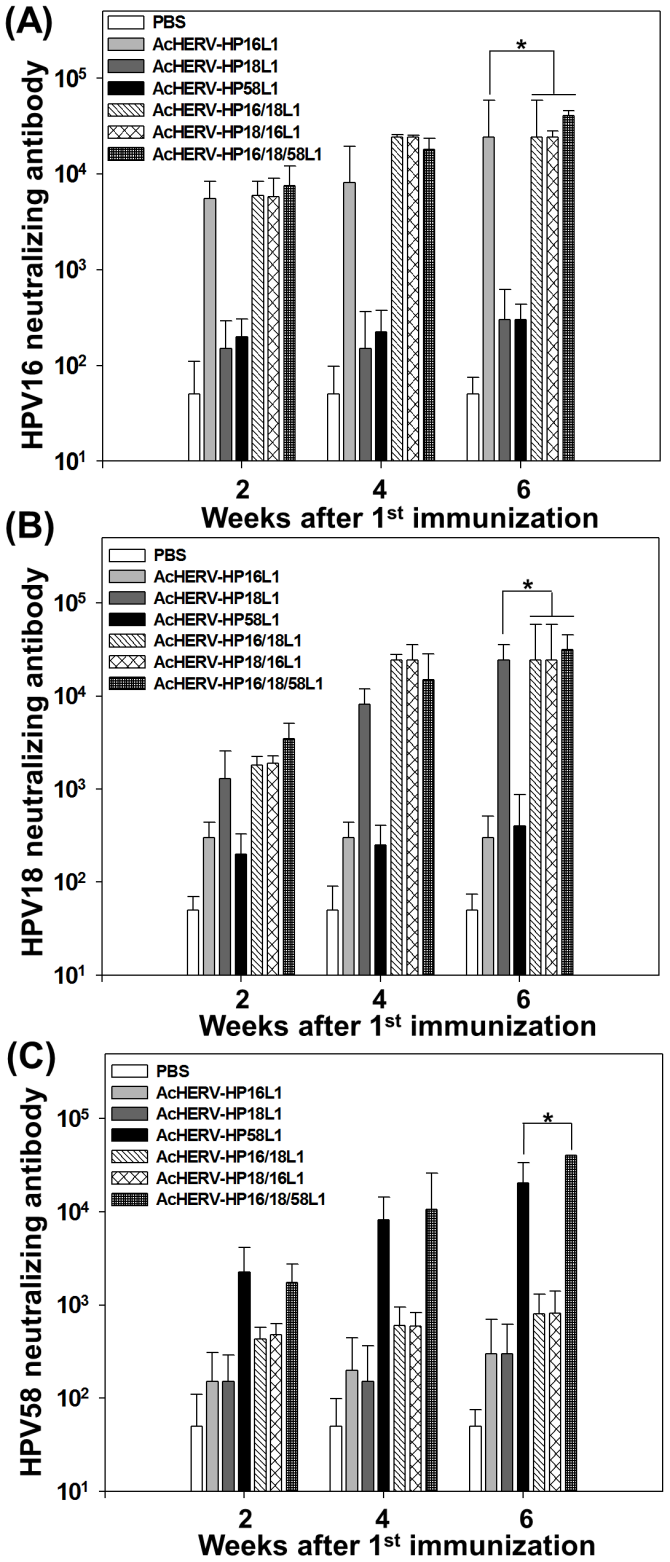
Induction of HPV type-specific neutralizing antibodies after immunization with AcHERV-based nanovaccines. Serum was sampled at 2, 4, 6 weeks after intramuscular administration with PBS or AcHERV-based nanovaccines. Neutralization assays were performed with HPV 16 (A) or HPV 18 (B) or HPV 58 (C) pseudoviruses. Data are expressed as the genometric means (log) of reciprocal serum dilutions that yielded a 50% reduction in SEAP. * p<0.05 (ANOVA and Student-Newman-Keuls test): Bivalent and trivalent groups were compared with each monovalent group.

Consistent with HPVL1-specific serum IgG and vaginal IgA induction data, neutralizing antibody induction capability and kinetics did not differ among groups administered monovalent, bivalent, or trivalent AcHERV-based nanovaccines. Six weeks after the first immunization, the titers of HPV 16 neutralizing antibodies were higher in groups treated with AcHERV-HP16L1, AcHERV-HP16/18L1, AcHERV-HP18/16L1, or AcHERV-HP16/18/58L1 compared to those in other groups (Fig. 6A). Titers of HPV 18 neutralizing antibodies were similar among groups treated with AcHERV-HP18L1, AcHERV-HP16/18L1, AcHERV-HP18/16L1, or AcHERV-HP16/18/58L1 (Fig. 6B). The titers of HPV58L1-specific (Fig. 6C) neutralizing antibodies were significantly higher in groups treated with AcHERV-HP58L1 or trivalent AcHERV-HP16/18/58 L1.

### Cell-mediated immune responses induced by AcHERV-based trivalent nanovaccines

**Figure 7:**
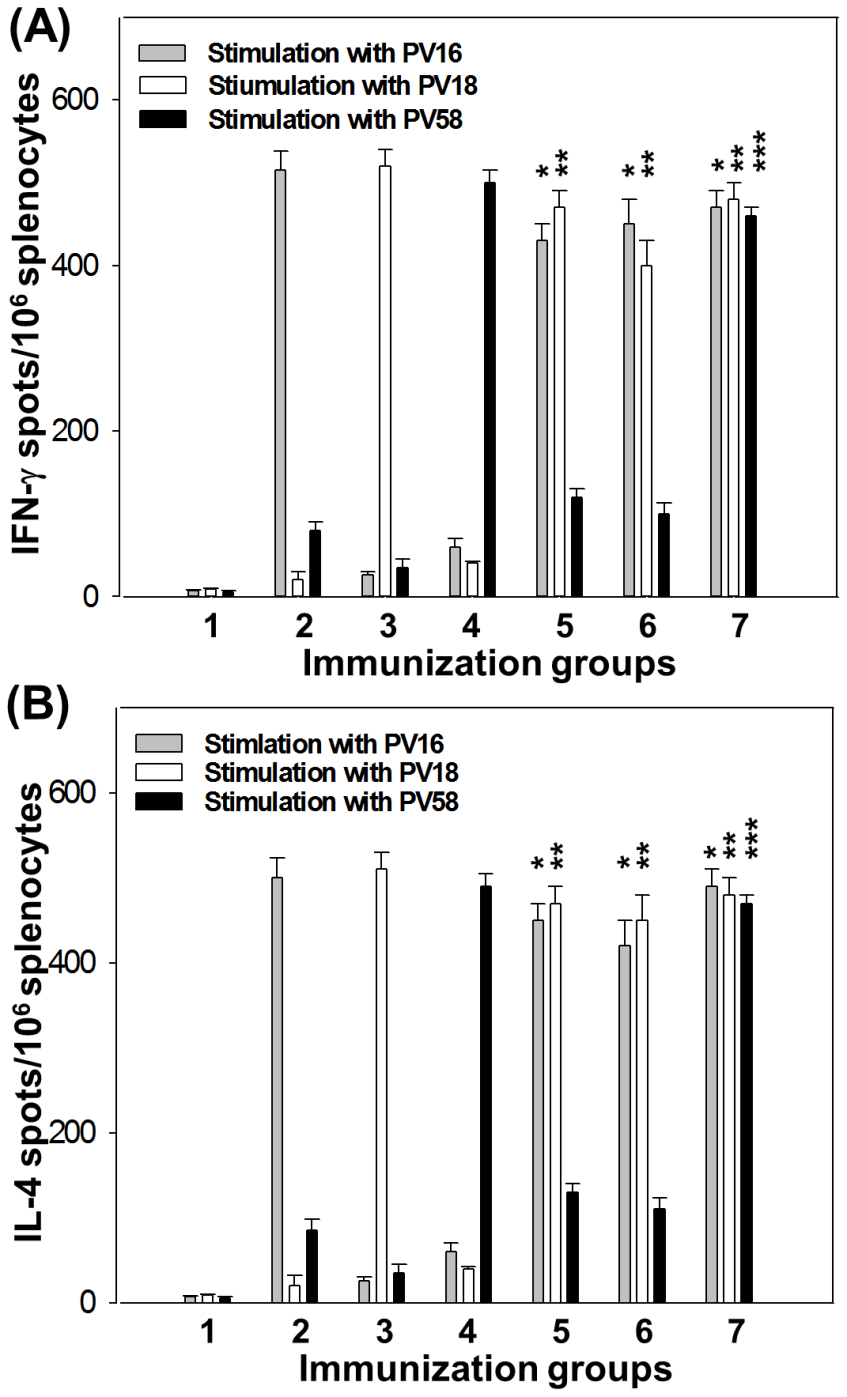
Induction of HPV 16, 18, and 58 L1-specific T cells. Mouse splenocytes were harvested 10 weeks after the first immunization. (A) The number of IFN-γ-producing HPV 16 or HPV 18 or HPV 58-specific CD8+ T cells was determined using an ELISPOT assay. (B) The number of IL-4-producing HPV 16 or HPV 18 or HPV 58-specific CD4+ T cell was determined using an ELISPOT assay. Values represent the number of spots per 10^6^ splenocytes following stimulation with HPV 16 or HPV 18 or HPV 58 pseudovirues (PV16, PV18, or PV58). Lane 1: PBS, Lane 2: AcHERV-HP16L1, Lane 3: AcHERV-HP18L1, Lane 4: AcHERV-HP58L1, Lane 5: AcHERV-HP16/18L1, Lane 6: AcHERV-HP18/16L1, Lane 7: AcHERV-HP16/18/58L1. * p<0.05 (ANOVA and Student-Newman-Keuls test): AcHERV-16L1 group was compared with bivalent or trivalent group. ** p<0.05 (ANOVA and Student-Newman-Keuls test): AcHERV-18L1 group was compared with bivalent or trivalent group. *** p<0.01 (ANOVA and Student-Newman-Keuls test): AcHERV-58L1 group was compared with trivalent group.

In addition to humoral immune responses, cell-mediated immune responses were induced by immunization with AcHERV-based nanovaccines (Fig. 7). Cellular immunity was determined by measuring IFN-γ (Th1) and IL-4 (Th2) in splenocytes from mice stimulated with trivalent, bivalent, or monovalent AcHERV-based nanovaccines. Similar to the humoral immune response data, the levels of IFN-γ (Fig. 7A) and IL-4 (Fig. 7B) produced by stimulated splenocytes were not significantly different among groups treated with trivalent, bivalent, or monovalent AcHERV-based nanovaccines. Intramuscular injection of mice with PBS did not induce the production of IFN-γ or IL-4.

### Protection of mice against challenge with HPV pseudoviruses by treatment with AcHERV-based multivalent nanovaccines

**Figure 8:**
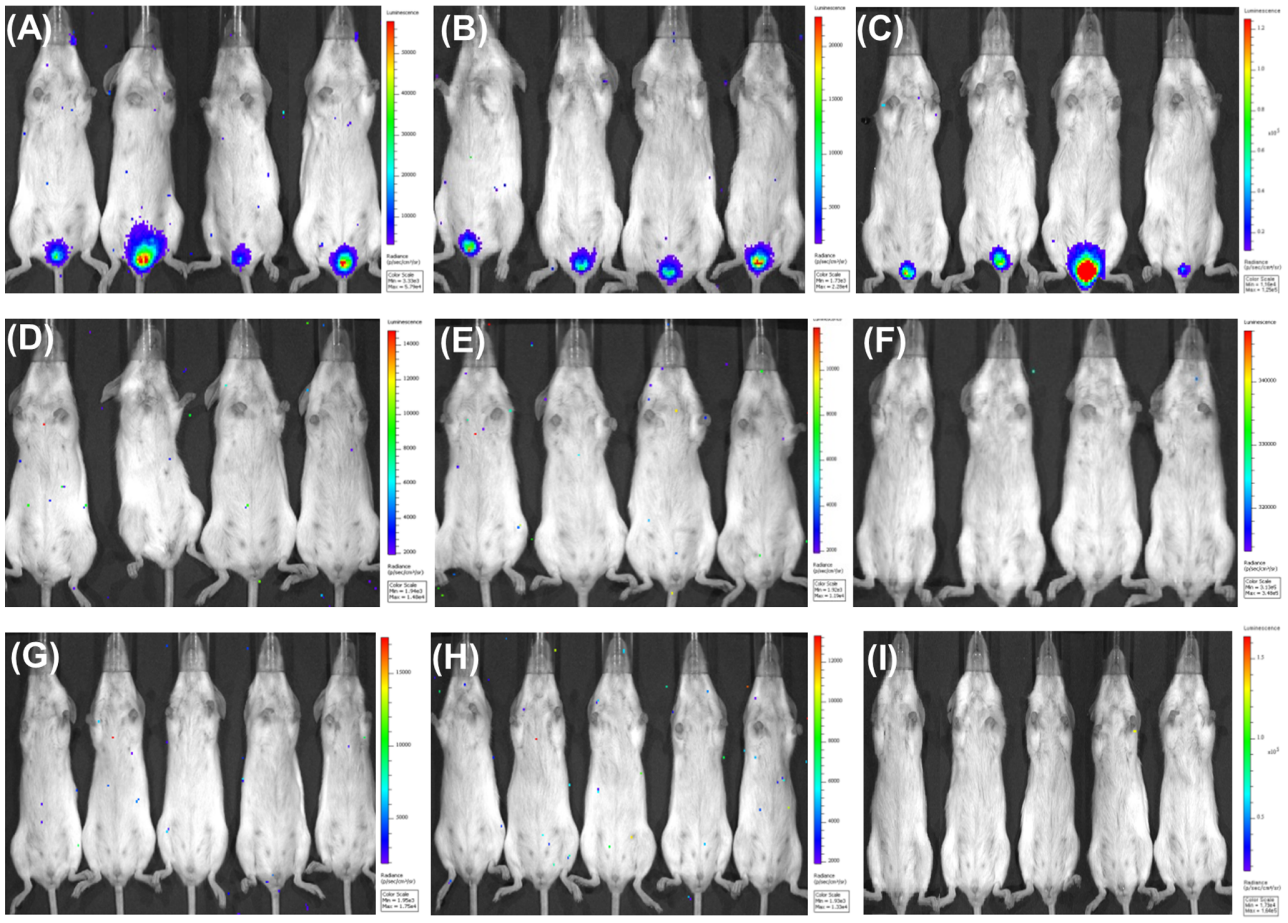
HPV challenge test with HPV pesudovirions in AcHERV-HPL1s immunized mice. Mice were immunized three times with PBS (A, B, C), AcHERV-HP16L1 (D), AcHERV-HP18L1 (E), AcHERV-HP58L1 (F), AcHERV-HP16/18L1 (G), AcHERV-HP18/16L1 (H), and AcHERV-HP16/18/58L1 (I) over 6 weeks. Ten weeks after the first immunization, mice received vaginal challenge with PV16 (A, D), PV18 (B, E), PV58 (C, F), the mixture of PV16 and PV18 (G, H), or with the mixture of PV16, PV18, and PV58 (I). Three days after challenge, mice were anesthetized, injected with luciferin, and the levels of luminescence were detected with an IVIS 200 bioluminescence imaging system.

To test whether IgG and IgA neutralizing antibody titers induced by AcHERV-based nanovaccines were sufficient to mediate protection in mice, we challenged AcHERV-based nanovaccine-treated mice with HPV 16, 18, and 58 pseudoviruses via the vaginal route. Vaginal pseudoinfection with HPV 16, 18, and 58 pseudoviruses was detected by monitoring the expression of the luciferase reporter gene using whole-organ, multispectral molecular imaging. Non-immunized mice challenged with HPV 16 (Fig. 8A), HPV 18 (Fig. 8B), or HPV 58 (Fig. 8C) pseudoviruses exhibited luciferase expression, reflecting effective vaginal pseudoinfection by pseudoviruses carrying the luciferase gene. In contrast, mice immunized with monovalent AcHERV-HP16L1 (Fig. 8D), AcHERV-HP18L1 (Fig. 8E), or AcHERV-HP58L1 (Fig. 8F) showed complete protection against homologous pseudovirus infection. Similar to results obtained with the monovalent nanovaccine, immunization with bivalent AcHERV-based nanovaccines (AcHERV-HP16/18L1 and AcHERV-HP18/16L1) provided complete protection against pseudoinfection by HPV 16 (Fig. 8G) or HPV 18 (Fig. 8H) pseudoviruses. Moreover, immunization with AcHERV-HP16/18/58L1 provided almost complete protection against pseudoinfection by mixtures of HPV 16, 18, and 58 pseudoviruses (Fig. 8I).

### Reactivities of various AcHERV-based nanovaccines

Table 2 shows the reactivities of various AcHERV-based nanovaccines to HPV 16, 18, and 58. Each titer is the mean value of IgG, IgA, neutralizing antibody, IFN-γ, and IL-4 in immunized mice. Measurements of serum IgG and vaginal IgA levels revealed slightly higher cross-reactivity to HPV 58 than HPV 18 in the group treated with AcHERV-HP16L1, and slightly higher cross-reactivity to HPV 16 than HPV 18 in the group treated with AcHERV-HP58L1. However, neutralizing antibody levels in the group treated with AcHERV-HP58L1 showed similarly low cross-reactivity to both HPV 16 and 18. The highest titers of IgG/IgA, neutralizing antibody, and cytokine levels obtained concurrently for HPV 16, 18, 58 were observed in the group treated with the trivalent AcHERV-based nanovaccine.

**Table 2:** Reactivity of AcHERV-based nanovaccines against HPV16, 18, and 58.

		Reactivity to each HPV type (%)					
Vaccines	HPV types	IgG^a^	IgA^a^	Neutralization^a^	IFN-γ^b^	IL-4^b^	
	HPV16	100	100	100	100	100	
Monovalent AcHERV-HP16L1	HPV18	5	4.8	1.2	5	5.2	
	HPV58	6.9	6.2	1.2	11.6	12	
	HPV16	4.1	9	1.2	3.8	3.9	
Monovalent AcHERV-HP18L1	HPV18	100	100	100	100	100	
	HPV58	5.1	3	1.6	7.7	7.8	
	HPV16	11.6	11.8	1.5	16	17.3	
Monovalent AcHERV-HP58L1	HPV18	4.7	3.1	1.5	6	7.1	
	HPV58	100	100	100	100	100	
	HPV16	100	74.7	100	85.4	87	
Bivalent AcHERV-HP16/18L1	HPV18	100	73.9	100	83.6	90.2	
	HPV58	5.5	19.7	4	22	24.5	
	HPV16	100	70.5	100	87.3	84	
Bivalent AcHERV-HP18/16L1	HPV18	100	70.7	100	76.9	88.2	
	HPV58	5.8	19.9	4	20	22.4	
	HPV16	100	87.1	100	91.2	98	
Trivalent AcHERV-HP16/18/58L1	HPV18	100	84.8	100	92.3	94.1	
	HPV58	93.4	67.7	100	92	95.9	

a IgG, IgA, and neutralization titers are serum dilution titer against each HPV pseudovirus and calculated with based on monovalent homologous titer. The 100% values indicate the 100% same to the titer values obtained for monovalent AcHERV-HPL1s (AcHERV-HP16L1, AcHERV-HP18L1, and AcHERV-HP58L1).

b IFN-γ and IL-4 are number of spots per 10^6^ splenocytes stimulated each pseudovirus HPV16, 18, and 58 and calculated with based on monovalent homologous spots. The 100% values indicate the 100% same to the spot numbers obtained for monovalent AcHERV-HPL1s.

## Discussion

In this study, we exploited the high gene capacity of baculoviral vectors, which can deliver DNAs up to ∼30 kbp in length, to construct the multivalent recombinant baculoviruses, AcHERV-HP16/18L1, AcHERV-HP18/16L1, and AcHERV-HP16/18/58L1, and compared their immunogenicity with those of monovalent AcHERV-HPL1s. The results obtained suggested no significant differences in transgene expression depending on the location of the gene in the baculoviral system. Importantly, bivalent (AcHERV-HP16/18L1 and AcHERV-HP18/16L1) and trivalent (AcHERV-HP16/18/58L1) AcHERV nanovaccines retained a degree of immunogenicity comparable to that of the three individual monovalent AcHERV-based nanovaccines.

Mice immunized with monovalent, bivalent, or trivalent AcHERV-HPL1 DNA vaccines exhibited similar high levels of IgG, IgA, and neutralizing antibody titers compared with monovalent AcHERV-HPL1 DNA vaccines. Among mice immunized with different AcHERV-based nanovaccines, those administered AcHERV-HP16/18/58L1 showed a greater induction of T cell immune responses, measured as IFN-γ and IL-4 levels, than mice treated with monovalent or bivalent AcHERV DNA vaccines. This observation suggests that our trivalent AcHERV-based nanovaccines elicited both Th2 and Th1 immune responses.

Notably, the trivalent AcHERV-based nanovaccine provided immune responses against HPV16, 18, and 58. Although the currently available prophylactic HPV vaccines Cervarix and Gardasil induce antibodies against HPV16 and 18, they do not provoke immune responses against HPV58. A recent, clinical study has shown that Cervarix provides no cross-protective immunity for HPV 58 [27]. It has been reported that HPV 58 is detected not only in cervical cancers, but also in high-grade squamous intraepithelial lesions (HISL) in women throughout Asia, Central America, and South America [5], [28]–[30]. Indeed, HPV 58 is known to be the second- and third-most common HPV type in Asian cases of cervical cancer and HSIL, respectively [31], [32]. A previous study reported that the genetic relationship between HPV 16 and 58 was closer than that between HPV 18 and 58 [27].

Understanding the important factors associated with vaccine efficacy is essential in developing and improving the next generation of vaccines. Vaccine efficacy may reflect a combination of a strong humoral response, cell-mediated response, and cross-reactivity characteristics. Cross-reactivity, in particular, is an important factor in vaccine development. Our cervicovaginal HPV pseudovirus challenge study revealed that AcHERV-based nanovaccines provided complete protection from each homologous pseudovirus (Fig. 8), but virtually no cross-reactivity with heterologous PVs (Table 2). Notably, the absence of neutralizing activity against HPV 58 psedovirus challenge by bivalent AcHERV-HP16/18L1 and AcHERV-HP18/16L1 emphasizes the lack of cross-reactive immune responses among HPV 16, 18, and 58 (Table 2). Cross-reactivity of an HPV 16/18 vaccine against HPV 31, 33, and/or 45 has been previously demonstrated [33], [34]; however, consistent with our observations, HPV 58 was found to show low cross-reactivity against an HPV 16/18 vaccine [35], [36].

In conclusion, the strong humoral and cell-mediated responses and virtual absence of cross-reactivity that follow immunization with multivalent AcHERV-HPL1s DNA vaccines suggest that these nanovaccines represent an efficient prophylactic treatment option that could contribute to global HPV prevention. Moreover, our AcHERV system provides a platform for future DNA antigen-encoding nanovaccines. Given the epidemiology of high-risk HPV types, the AcHERV-based HPV 16/18/58 trivalent nanovaccine could be clinically useful for the prevention of HPV transmission in Asia.

## Materials and Methods

### Cell culture

Spodoptera frugiperda 9 (Sf9) insect cells were propagated at 28°C in Sf-900 II medium (Invitrogen, Carlsbad, CA, USA) supplemented with antibiotics and antimycotics (Invitrogen). 293TT cells were cultured in Dulbecco's modified Eagle medium (DMEM) supplemented with 10% fetal bovine serum (FBS), hygromycin B (400 µg/ml; Invitrogen) and 1% penicillin/streptomycin (Gibco, Grand Island, NY, USA).

### Ethics

Six-week-old female BALB/c mice were purchased from Orient-Bio (Seungnam, Kyonggi-do, Korea) and housed in filter-top cages, with water and food provided *ad libitum*. Mice were maintained in accordance with the Guide for the Care and Use of Laboratory Animals of Konkuk University (Seoul, Republic of Korea), and were housed in a Bio-safety Level 2 facility. The use of animals in these experiments was approved by the Institutional Animal Care and Use Committee of Konkuk University (Approval No. KU12078). Throughout the study, the condition of the animals was monitored twice a day. In this study, no mice exhibited symptoms of illness or appeared to be close to death. Moreover, no mice died during the monitoring phase. After final monitoring, mice were humanely euthanized using cervical dislocation according to the AVMA guidelines for the euthanasia of animals.

### Construction of transfer plasmids

The recombinant baculoviral trivalent HPV nanovaccine was constructed by introducing the HPV16/18/58L1 genes from p16L1/L2, p18L1/L2, and p58L1/L2 (kindly supplied by Dr. Schiller, National Cancer Institute, National Institutes of Health, USA) into the baculovirus vector under the control of the human elongation factor 1 promoter. Two dual-gene cassettes, one encoding the HPV 16L1 sequence ahead of HPV 18L1 (pFB-HP16/18L1) and one encoding the HPV 18L1 sequence ahead of HPV 16L1 (pFB-HP18/16L1), were constructed by cloning into pFastBac1. pFB-HP16/18/58L1 was constructed by subcloning the HPV 58L1 gene into the *Not*I site of pFB-HP16/18L1. Then, a codon-optimized envelope gene of human endogenous retrovirus (HERV; GenBank accession number NM014590; GenScript Corp., Piscataway, NJ, USA) was inserted into the *Eco*RI site of pFB-HP16/18L1, pFB-HP18/16L1, and pFB-HP16/18/58L1. Schematic diagrams of pFB-HERV-HP16/18L1, pFB-HERV-HP18/16L1, and pFB-HERV-HP16/18/58L1 are shown in Figure 1.

### Construction of recombinant baculoviruses

Recombinant baculoviruses were produced using the Bac-to-Bac baculovirus expression system (Invitrogen) according to the manufacturer's instructions. The recombinant baculovirus encoding the HPV 16L1 sequence ahead of 18L1 was designated AcHERV-HP16/18L1; the recombinant baculovirus encoding the HPV 18L1 sequence ahead of 16L1 was designated AcHERV-HP18/16L1; and the recombinant baculovirus encoding the HPV 16L1 sequence followed by 18 L1 and 58 L1 was designated AcHERV-HP16/18/58L1. Recombinant AcHERV-HP16/18L1, AcHERV-HP18/16L1, and AcHERV-HP16/18/58L1 baculoviruses were further amplified by propagation in Sf9 cells. Baculoviruses were purified by first centrifuging at 2000× g at 4°C for 10 minutes to remove virus-infected cell debris, after which supernatants were overlaid on a 30% sucrose cushion and centrifuged at 35,000 rpm at 4°C for 1.5 hour in a 50.2Ti rotor (Beckman Coulter Inc., Brea, CA, USA). The pellet was re-suspended in phosphate-buffered saline (PBS) and used for immunization.

### Characterization of recombinant baculoviruses

HPV L1s and HERV genes from recombinant baculovirus were analyzed by polymerase chain reaction (PCR; GeneAmp PCR system 9700; Perkin-Elmer Cetus, Chandler, AZ, USA) using viral DNA extracted with the NucleoSpin RNA Virus kit (Macherey-Nagal, Germany). The expression of HPV 16L1, 18L1, and 58L1 genes was detected by transducing human 293TT cells with each constructed recombinant baculovirus at a multiplicity of infection (MOI) of 100. After a 48-hour incubation, total RNA was extracted from 293TT cells using an RNeasy mini kit (Qiagen, Valencia, CA, USA) and cDNA was synthesized using M-MuLV reverse transcriptase (Bioneer, Daejeon, Republic of Korea). The cDNA was amplified by PCR using the following primer pairs: HPV 16L1, 5′-CAGCGAGACCACCTACAAGA-3′ (forward) and 5′-GCTGTTCATGCTGTGGATGT-3′ (reverse), generating a 139-bp product; HPV 18L1, 5′-ACCAGGTCCACAAACCTGAC-3′ (forward) and 5′-GGAGGAGTTCATGGAGTGGA-3′ (reverse), generating a 188-bp product, and HPV 58L1, 5′-AGGAGGGCACCTACAAGAACGA-3′ (forward) and 5′-GAACCTGTAGGTGTCCTGCAGG-3′ (reverse), generating a 209-bp product. 18s ribosomal RNA was used as an internal control for the efficiency of RT-PCR among samples. PCR was carried out using an initial denaturation step at 94°C for 3 minutes followed by 30 cycles of 30 seconds at 94°C, 20 seconds at 62°C, and 20 seconds at 72°C.

### Western blotting

Western blotting was used to test the expression of HPV 16L1, 18L1, and 58L1 proteins in cells after delivery of the corresponding AcHERV-HPL1s. 293TT cells were treated with each recombinant baculovirus at a MOI of 100. Three days after infection, cells were lysed with lysis buffer, and proteins in lysates were resolved by sodium dodecyl sulfate-polyacrylamide gel electrophoresis (SDS-PAGE) and transferred to nitrocellulose membranes. HPV L1 protein was detected by first incubating the membranes with primary anti-HPV16 L1 antibody (Camvir-1; Santa Cruz Biotechnology, Santa Cruz, CA, USA), and polyclonal mouse sera specific for HPV 18 or 58 prepared in our laboratory. An anti-β-actin antibody (1∶2,000 dilution; Santa Cruz Biotechnology) was used as a protein loading control.

### Immunization of mice with AcHERV-HPL1s

Six-week-old female BALB/c mice were immunized by intramuscular injection into the hind legs with 1×10^7^ plaque-forming units (PFU) of AcHERV-HPL1s. Sixty BALB/c mice were divided into seven groups (Table 1). Mice were immunized three times with AcHERV-HPL1s at 2-week intervals. Sera and vaginal washes from each group were obtained 0, 2, 4, and 6 weeks after the first immunization. Blood samples and vaginal washings were collected after anesthetizing the mice by intramuscular injection with 40mg/kg of Zoletil50 (Virbac Laboratories, Carros, France), and 51 mg/kg of Rompun (Bayer Korea, Seoul, Republic of Korea). Blood was sampled from right external jugular vein, and vaginal samples were collected by rinsing out the vaginal cavity with 50 ul of PBS 5 times. Vaginal washing samples were then centrifuged at 13,000 rpm for 10 min, and the supernatants were used for further assays.

### Generation of the HPV 16, 18, and 58 pseudoviruses

HPV 16, 18, and 58 pseudoviruses were prepared by co-transfection of 293TT cells with pSEAP (secreted alkaline phosphatase) or pLucf (luciferase) marker plasmid together with p16L1/L2, p18L1/L2, or p58L1/L2 plasmids. After incubation at 37°C for 48 hours, cells were lysed by adding Triton X-100 (Sigma, St. Louis, MO, USA) at a final concentration of 0.5% in Dulbecco's phosphate-buffered saline supplemented with 9.5 mM MgCl_2_. Lysates were digested with 0.2% Benzonase (Sigma) for 24 hours at 37°C to complete virus maturation. Lysates were mixed with 0.8 M NaCl and clarified by centrifugation at 2,000× g for 15 minutes. Pseudoviruses were purified on OptiPrep density gradient medium (Sigma) by centrifugation at 234,000× g for 4 hours. After centrifugation, fractions were collected and stored at -80°C.

### Enzyme-linked immunosorbent assay

Production of HPV 16-, 18-, and 58L1-specific antibodies was tested by enzyme-linked immunosorbent assay (ELISA) using pseudoviruses as coating antigens. Sixty microliters of pseudovirus (0.001 mg/ml) was added to each well of a 96-well plate and incubated for 16 hours at 4°C. After washing, plates were blocked with 2% (w/v) bovine serum albumin in PBS containing 0.1% Nonidet P-40 (Sigma). Serially diluted mouse sera or vaginal washings (60 ul/well) were added and incubated at room temperature for 2 hours. After washing, peroxidase-conjugated goat anti-mouse IgG antibody (1∶2000; Santa Cruz Biotechnology) or goat anti-mouse IgA antibody (1∶1000; Santa Cruz Biotechnology) was added. For color development, 1-Step Turbo TMB (3,39,5,59-tetramethyl benzidine substrate solution; Pierce, Rockford, IL, USA) was added. Endpoint titers were defined as the highest serum dilutions that resulted in an absorbance value twice that of non-immunized serum (cutoff value, 0.1) and were expressed as group means ± SDs.

### Neutralization assay

Neutralizations assays were performed using SEAP-expressing pseudoviruses (PV16, PV18, and PV58) according to a previously described method [24]. Briefly, OptiPrep-purified, SEAP-expressing HPV 16, 18, and 58 pseudoviruses were diluted 3,000-fold and incubated on ice for 1 hour with 3-fold serial dilutions of mouse sera. 293TT cells were infected by incubating with each pseudovirus–antibody mixture for 3 days. The SEAP content in 10 ul of clarified cell supernatant was determined using a Great EscAPe SEAP Chemiluminescence Kit (Clontech, Mountain View, CA, USA). Neutralization titers were defined as the reciprocal of the highest serum dilution that caused at least a 50% reduction in SEAP activity.

### IFN-γ/IL-4 enzyme-linked immunospot assay

The production of interferon (IFN-γ) and interleukin 4 (IL-4) from splenocytes of immunized mice was detected by enzyme-linked immunospot (ELISPOT) assay. A 96-well plate was coated with 0.2 ug of anti-mouse IFN-γ and anti-mouse IL-4 capture antibodies, and then blocked by incubating with 10% FBS at 37°C. Splenocytes were seeded at 1×10^6^ cells per well in 100 ul of medium, and stimulated by adding 1×10^6^ HPV pseudoviruses and incubating for an additional 24 hours at 37°C. Plates were then washed with PBS containing 0.05% Tween-20 and treated with 20 ng of biotinylated anti-mouse IFN-γ and anti-mouse IL-4 detection antibodies. After 2 hours, streptavidin-alkaline phosphatase was added to the wells, and color was developed with an AEC substrate reagent (BD Biosciences, Franklin Lakes, NJ, USA). The number of spots was counted using an ELISPOT reader (AID Elispot Reader ver. 4; Strassberg, Germany).

### Challenge test with HPV pseudoviruses

Six weeks after the final immunization with AcHERV-HPL1s (AcHERV-HP16L1, AcHERV-HP18L1, AcHERV-HP58L1, AcHERV-HP16/18L1, ACHERV-HP18/16L1, or AcHERV-HP16/18/58L1), mice were challenged with HPV pseudoviruses, as described previously [24] Seven days before *in vivo* genital challenge with pseudoviruses, mice were synchronized in a diestrus-like status by subcutaneous injection of 3 mg DepoProvera (Pfizer AG, Zurich, Switzerland). Six hours prior to pseudovirus challenge, mice were deeply anesthetized by intramuscular injection with 40 mg/kg of Zoletil50 (Virbac Laboratories), and 5 mg/kg of Rompun (Bayer Korea), then pretreated intravaginally with 20 ul of 4% nonoxynol-9 (Sigma). Mice were genitally challenged with 1×10^7^ IFU of HPV 16, 18, and 58 pseudoviruses, each in a 20 ul solution containing 2% carboxymethylcellulose (Sigma). Three days later, all mice were anesthetized by intramuscular injection with 40 mg/kg of Zoletil50 (Virbac Laboratories), and 5 mg/kg of Rompun (Bayer Korea). Anesthetized mice were injected intraperitoneally with luciferin (30 ul at 7 mg/ml) to detect luciferase expressed upon pseudoinfection by pseudoviruses encapsidating pLucf, a plasmid carrying the luciferase gene [http://home.ccr.cancer.gov/lco/]. The expression of luciferase was detected by measuring light emission over 10 minutes with an IVIS 200 bioluminescence imaging system (Xenogen, Cranbury, NJ, USA). Equal-sized areas encompassing the site of virus inoculation were analyzed using Living Image 2.20 software (Xenogen) [37]. Due to the lack of pathogenicity of pseudotype HPV virus, all the pseudovirus-challenged mice survived until the end of the study, and did not show any signs of illness such as ruffled fur, decreased activity, and weight loss regardless of vaccination. After final monitoring, all the challenged mice were humanely euthanized using cervical dislocation according to the AVMA guidelines for the euthanasia of animals.

### Statistical analysis

All data were analyzed by analysis of variance (ANOVA) with Student-Newman-Keuls post hoc tests using SigmaStat software (Systat Software, Richmond, CA, USA). P-values less than 0.05 were considered significant.

## References

[1] zur Hausen H (2002) Papillomaviruses and cancer: from basic studies to clinical application. Nature reviews Cancer 2: 342–350. 12044010 10.1038/nrc798

[2] Dell G, Gaston K (2001) Human papillomaviruses and their role in cervical cancer. Cellular and molecular life sciences: CMLS 58: 1923–1942. 11766888 10.1007/PL00000827 PMC11337310

[3] Daftarian P, Mansour M, Benoit AC, Pohajdak B, Hoskin DW, et al (2006) Eradication of established HPV 16-expressing tumors by a single administration of a vaccine composed of a liposome-encapsulated CTL-T helper fusion peptide in a water-in-oil emulsion. Vaccine 24: 5235–5244. 16675074 10.1016/j.vaccine.2006.03.079

[4] Ault KA, Giuliano AR, Edwards RP, Tamms G, Kim LL, et al (2004) A phase I study to evaluate a human papillomavirus (HPV) type 18 L1 VLP vaccine. Vaccine 22: 3004–3007. 15297048 10.1016/j.vaccine.2004.02.020

[5] Chan PK, Li WH, Chan MY, Ma WL, Cheung JL, et al (1999) High prevalence of human papillomavirus type 58 in Chinese women with cervical cancer and precancerous lesions. Journal of medical virology 59: 232–238. 10459162

[6] Chan PK (2012) Human papillomavirus type 58: the unique role in cervical cancers in East Asia. Cell & bioscience 2: 17. 22571619 10.1186/2045-3701-2-17 PMC3414832

[7] Kuck D, Lau T, Leuchs B, Kern A, Muller M, et al (2006) Intranasal vaccination with recombinant adeno-associated virus type 5 against human papillomavirus type 16 L1. Journal of virology 80: 2621–2630. 16501072 10.1128/JVI.80.6.2621-2630.2006 PMC1395428

[8] Ferraro B, Morrow MP, Hutnick NA, Shin TH, Lucke CE, et al (2011) Clinical applications of DNA vaccines: current progress. Clinical infectious diseases: an official publication of the Infectious Diseases Society of America 53: 296–302. 21765081 10.1093/cid/cir334 PMC3202319

[9] Liu MA (2010) Immunologic basis of vaccine vectors. Immunity 33: 504–515. 21029961 10.1016/j.immuni.2010.10.004

[10] Bodles-Brakhop AM, Draghia-Akli R (2008) DNA vaccination and gene therapy: optimization and delivery for cancer therapy. Expert review of vaccines 7: 1085–1101. 18767956 10.1586/14760584.7.7.1085

[11] Bolhassani A, Safaiyan S, Rafati S (2011) Improvement of different vaccine delivery systems for cancer therapy. Molecular cancer 10: 3. 21211062 10.1186/1476-4598-10-3 PMC3024302

[12] Chen CY, Liu HJ, Tsai CP, Chung CY, Shih YS, et al (2010) Baculovirus as an avian influenza vaccine vector: differential immune responses elicited by different vector forms. Vaccine 28: 7644–7651. 20883735 10.1016/j.vaccine.2010.09.048

[13] Cho HJ, Han SE, Im S, Lee Y, Kim YB, et al (2011) Maltosylated polyethylenimine-based triple nanocomplexes of human papillomavirus 16L1 protein and DNA as a vaccine co-delivery system. Biomaterials 32: 4621–4629. 21440296 10.1016/j.biomaterials.2011.03.004

[14] Best SR, Peng S, Juang CM, Hung CF, Hannaman D, et al (2009) Administration of HPV DNA vaccine via electroporation elicits the strongest CD8+ T cell immune responses compared to intramuscular injection and intradermal gene gun delivery. Vaccine 27: 5450–5459. 19622402 10.1016/j.vaccine.2009.07.005 PMC2745985

[15] Ohlschlager P, Spies E, Alvarez G, Quetting M, Groettrup M (2011) The combination of TLR-9 adjuvantation and electroporation-mediated delivery enhances in vivo antitumor responses after vaccination with HPV-16 E7 encoding DNA. International journal of cancer Journal international du cancer 128: 473–481. 20309939 10.1002/ijc.25344

[16] Brandsma JL, Shlyankevich M, Zhang L, Slade MD, Goodwin EC, et al (2004) Vaccination of rabbits with an adenovirus vector expressing the papillomavirus E2 protein leads to clearance of papillomas and infection. Journal of virology 78: 116–123. 14671093 10.1128/JVI.78.1.116-123.2004 PMC303402

[17] Kaufmann AM, Stern PL, Rankin EM, Sommer H, Nuessler V, et al (2002) Safety and immunogenicity of TA-HPV, a recombinant vaccinia virus expressing modified human papillomavirus (HPV)-16 and HPV-18 E6 and E7 genes, in women with progressive cervical cancer. Clinical cancer research: an official journal of the American Association for Cancer Research 8: 3676–3685. 12473576

[18] Cho HJ, Oh YK, Kim YB (2011) Advances in human papilloma virus vaccines: a patent review. Expert opinion on therapeutic patents 21: 295–309. 21250872 10.1517/13543776.2011.551114

[19] Lee HJ, Park N, Cho HJ, Yoon JK, Van ND, et al (2010) Development of a novel viral DNA vaccine against human papillomavirus: AcHERV-HP16L1. Vaccine 28: 1613–1619. 19961961 10.1016/j.vaccine.2009.11.044

[20] Kost TA, Condreay JP (2002) Recombinant baculoviruses as mammalian cell gene-delivery vectors. Trends in biotechnology 20: 173–180. 11906750 10.1016/s0167-7799(01)01911-4

[21] Lung O, Westenberg M, Vlak JM, Zuidema D, Blissard GW (2002) Pseudotyping Autographa californica multicapsid nucleopolyhedrovirus (AcMNPV): F proteins from group II NPVs are functionally analogous to AcMNPV GP64. Journal of virology 76: 5729–5736. 11992001 10.1128/JVI.76.11.5729-5736.2002 PMC137061

[22] Wilson S, Baird M, Ward VK (2008) Delivery of vaccine peptides by rapid conjugation to baculovirus particles. Vaccine 26: 2451–2456. 18417258 10.1016/j.vaccine.2008.03.027

[23] Buck CB, Pastrana DV, Lowy DR, Schiller JT (2004) Efficient intracellular assembly of papillomaviral vectors. Journal of virology 78: 751–757. 14694107 10.1128/JVI.78.2.751-757.2004 PMC368835

[24] Lee HJ, Hur YK, Cho YD, Kim MG, Lee HT, et al (2012) Immunogenicity of bivalent human papillomavirus DNA vaccine using human endogenous retrovirus envelope-coated baculoviral vectors in mice and pigs. PloS one 7: e50296. 23209698 10.1371/journal.pone.0050296 PMC3507738

[25] Choi JY, Gwon YD, Kim JK, Cho YD, Heo YK, et al (2013) Protective Efficacy of a Human Endogenous Retrovirus Envelope-Coated, Nonreplicable, Baculovirus-Based Hemagglutin Vaccine against Pandemic Influenza H1N1 2009. PloS one 8: e80762. 24260476 10.1371/journal.pone.0080762 PMC3832454

[26] Nieto K, Stahl-Hennig C, Leuchs B, Muller M, Gissmann L, et al (2012) Intranasal vaccination with AAV5 and 9 vectors against human papillomavirus type 16 in rhesus macaques. Human gene therapy 23: 733–741. 22401308 10.1089/hum.2011.202 PMC3404423

[27] Wheeler CM, Castellsague X, Garland SM, Szarewski A, Paavonen J, et al (2012) Cross-protective efficacy of HPV-16/18 AS04-adjuvanted vaccine against cervical infection and precancer caused by non-vaccine oncogenic HPV types: 4-year end-of-study analysis of the randomised, double-blind PATRICIA trial. The lancet oncology 13: 100–110. 22075170 10.1016/S1470-2045(11)70287-X

[28] Hara H, Honda A, Suzuki H, Sata T, Matsukura T (2004) Detection of human papillomavirus type 58 in polydactylous Bowen's disease on the fingers and toes of a woman - concurrent occurrence of invasive vulval and cervical carcinomas. Dermatology 209: 218–222. 15459536 10.1159/000079893

[29] Bao YP, Li N, Smith JS, Qiao YL (2008) Human papillomavirus type distribution in women from Asia: a meta-analysis. International journal of gynecological cancer: official journal of the International Gynecological Cancer Society 18: 71–79. 10.1111/j.1525-1438.2007.00959.x 17466054

[30] Zhang T, Xu Y, Qiao L, Wang Y, Wu X, et al (2010) Trivalent Human Papillomavirus (HPV) VLP vaccine covering HPV type 58 can elicit high level of humoral immunity but also induce immune interference among component types. Vaccine 28: 3479–3487. 20211219 10.1016/j.vaccine.2010.02.057

[31] Clifford G, Franceschi S, Diaz M, Munoz N, Villa LL (2006) Chapter 3: HPV type-distribution in women with and without cervical neoplastic diseases. Vaccine 24 Suppl 3 S3/26–34. 10.1016/j.vaccine.2006.05.026 16950015

[32] Kwag HL, Kim HJ, Chang DY (2012) The production and immunogenicity of human papillomavirus type 58 virus-like particles produced in Saccharomyces cerevisiae. J Microbiol 50: 813–820. 23124750 10.1007/s12275-012-2292-1

[33] Bonanni P, Boccalini S, Bechini A (2009) Efficacy, duration of immunity and cross protection after HPV vaccination: a review of the evidence. Vaccine 27 Suppl 1 A46–53. 19480962 10.1016/j.vaccine.2008.10.085

[34] Kemp TJ, Hildesheim A, Safaeian M, Dauner JG, Pan Y, et al (2011) HPV16/18 L1 VLP vaccine induces cross-neutralizing antibodies that may mediate cross-protection. Vaccine 29: 2011–2014. 21241731 10.1016/j.vaccine.2011.01.001 PMC3046309

[35] Herrero R (2009) Human papillomavirus (HPV) vaccines: limited cross-protection against additional HPV types. The Journal of infectious diseases 199: 919–922. 19236278 10.1086/597308

[36] Scherpenisse M, Schepp RM, Mollers M, Meijer CJ, Berbers GA, et al (2013) Characteristics of HPV-Specific Antibody Responses Induced by Infection and Vaccination: Cross-Reactivity, Neutralizing Activity, Avidity and IgG Subclasses. PloS one 8: e74797. 24058629 10.1371/journal.pone.0074797 PMC3776846

[37] Roberts JN, Buck CB, Thompson CD, Kines R, Bernardo M, et al (2007) Genital transmission of HPV in a mouse model is potentiated by nonoxynol-9 and inhibited by carrageenan. Nature medicine 13: 857–861. 10.1038/nm1598 17603495

